# Narrative review of human milk fortifiers for preterm infants

**DOI:** 10.3389/fped.2026.1814184

**Published:** 2026-06-29

**Authors:** Lu Wang, Dingding Yin, Qin Tang, Ping Zhou

**Affiliations:** 1 Department of Pediatrics, The First Affiliated Hospital of Jinan University, Guangzhou, China; 2 Department of Neonatology, Baoan Women's and Children's Hospital, Shenzhen, China

**Keywords:** extrauterine growth restriction, human milk fortifier, multi-component fortifiers, preterm infants, single-component fortifier

## Abstract

The advancements in perinatal medicine and neonatal intensive care have enhanced the survival rates of extremely preterm infants. Nevertheless, the incidence of extrauterine growth restriction persists at a relatively high level. As the optimal enteral feeding source for preterm infants, human milk alone may be unable to fulfill the high nutritional requirements of very preterm infants. Consequently, the supplementation of human milk with nutritional fortifiers has become a widely recognized practice. At present, diverse types of human milk fortifiers are employed in different enteral feeding situations for preterm infants. This article provides a descriptive review of the current use of various fortifiers and also introduces novel or investigational fortifiers.

## 1 Introduction

Annually, approximately 15 million preterm infants are born worldwide, constituting 9.9% of all live births, and around 15% of them are very preterm infants born before 32 weeks of gestation (1). In comparison to full-term infants, very preterm infants do not experience the intrauterine nutritional accumulation process during the later stages of pregnancy. As a result, they require elevated levels of protein, energy, fatty acids, minerals, and micronutrients after birth for optimal-growth. Human milk remains the optimal nutritional source for premature infants with immature immune and intestinal functions. Nevertheless, exclusive human milk alone may be unable to fulfill all nutritional requirements (2, 3). To reduce the risk of cognitive impairment (4) and other adverse consequences related to extrauterine growth restriction [EUGR, which is defined as anthropometrics at discharge lower or equal to the 10th percentile for corrected age (5)], supplementing human milk with fortifiers (human milk fortifier, HMF) to optimise protein, energy, minerals, and other nutrients has become a global standard practice in the enteral nutritional management of premature infants (6).

To meet the evolving clinical demands, HMFs have been continuously improved since their introduction fifty years ago (7). In 1995, more advanced formulations containing sodium, iron, zinc, and vitamins emerged (8), and currently, the variety of fortifiers is on the rise. Although some neonatal intensive care units (NICUs) in the United States employ fortifiers derived from donated human milk, multi-component fortifiers sourced from cow's milk still serve as the primary approach for human milk fortification in clinical practice (9). Considering that a substantial proportion of preterm infants still present with EUGR after standard fortification with multi-component fortifiers (assuming the protein content and energy density of breast milk remain unchanged), relevant clinical studies have shown that the majority of preterm infants receiving standard fortification fail to achieve recommended protein intake, and their growth rate lags behind that of intrauterine fetuses of the same gestational age (10). One observational study found that after standard fortification, 58% of very preterm infants still exhibited EUGR at discharge (11). A Chinese multi-center survey yielded similar results, reporting an EUGR incidence of 60.7% among very preterm infants (12). Many NICUs have started adding single-component fortifiers to the standard fortification regimen to increase the protein content of breast milk and elevate caloric levels (13). The extensive use of these fortifiers has enhanced the nutritional intake of preterm infants and promoted their growth and development (14, 15). Although there is no strong evidence to establish a causal relationship between fortification and complications (16), it has been observed that the addition of fortifiers may be associated with feeding intolerance, necrotizing enterocolitis (NEC) (9).

In response to these concerns, researchers are constantly refining existing fortifiers or exploring novel sources. This paper examines the clinical advantages and limitations of these fortifiers while introducing emerging alternatives, thereby enhancing neonatal healthcare professionals’ awareness of the human milk fortification landscape.

The literature review included electronic searches of PubMed (1997-31 July 2025), EMBASE (1974-31 July 2025), Web of Science (1997-31 July 2025), and the China National Knowledge Infrastructure (1999-31 July 2025), Wan Fang (1993-31 July 2025), and China Science and Technology Journal databases (1989-31 July 2025), using the keywords ‘preterm infant’ or‘neonate’ and ‘fortifier’ or ‘fortification’, by one independent reviewer. English and Chinese articles were included. The search included randomized controlled trials, cohort studies, systematic reviews and meta-analysis. Articles lacking detailed information and case reports were excluded.

## 2 The necessity of human milk fortification for preterm infants

Beyond its content of macronutrients and micronutrients, breast milk represents a naturally abundant source of multiple crucial bioactive components, including immunoglobulins, bioactive peptides, and growth factors. These components can notably reduce the all-cause mortality rate and the incidence of various complications in preterm infants, while concurrently enhancing long-term neurodevelopmental and cardiovascular health (17, 18). The protein content in the breast milk of mothers with preterm infants may decline from 1.4–1.5 g/dL during the initial two weeks of lactation to approximately 1 g/dL by weeks 4–6 (10, 19), with substantial variation noted among different mothers (15, 20). The protein-to-energy ratio (P/E) in unfortified human milk is approximately 1.8 g/100 kcal, significantly lower than the recommended 3.2–4.1 g/100 kcal necessary to support optimal protein utilization and physical growth in preterm infants (14, 15, 21). Breast milk with a feeding volume of 150–180 mL/kg/day also supplies less energy than the recommended intake of 110–135 kcal/kg/day required to meet the growth demands of preterm infants (14). Additionally, various micronutrients are also in short supply, and there are substantial regional disparities in the micronutrient content of breast milk across different resource settings (22, 23). When a mother's own milk is inadequate or unavailable, donor milk is typically chosen as a supplement or substitute. Donor milk mainly stems from donations by mothers of term infants several months postpartum, and it has even lower energy and protein concentrations (24).

Consequently, exclusive human milk alone may be unable to provide sufficient protein, energy, and other micronutrients for preterm infants, rendering it challenging to attain the ideal intrauterine-like growth rate (25, 26). This nutritional deficiency results in EUGR in preterm infants, heightening the risk of neurocognitive impairment and other adverse health outcomes (24, 26–28). As a result, fortifying human milk with supplements to augment its nutritional content has become a widely recognized clinical practice, which supports improved bone mineralization, linear growth, and neurocognitive development in preterm infants (13, 15).

## 3 Classification of human milk fortifiers

A diverse array of products exists for the fortification of human milk intended for preterm infants, which vary in terms of milk source and nutritional composition.

### 3.1 Classification based on milk source

Currently available HMFs predominantly utilize cow's milk as their source. In North America, certain NICUs employ fortifiers derived from donor milk, while in specific resource-constrained regions, preterm infant formula is utilized as a fortifier. Fortifiers derived from donkey milk or bovine colostrum are still in the research stage (29) (Table 1).

**Table 1:** Clinical studies on human milk fortifiers derived from different sources.

Fortifier	Reference	Study type	Sample size	Gestation age	Result	Conclusion
Cows milk-based	Zachariassen, et al, 2011 (41)	RCT	324	24–32 weeks	Increased the risk of developing atopic dermatitis and recurrent wheezing. Boys had an increased risk of developing recurrent wheezing	Cows milk-based fortification for preterm infants was not associated with an increased risk of allergic diseases up to 1 year of age
	Vaks, et al, 2018 (42)	Poisson regression analysis	139	<35 weeks	The DERR for NEC onset was significantly greater on days for which infants were <14 days of age or ≥31 weeks postmenstrual age, or which fell within 14 days after initiation of enteric feeding or 4 days after introduction of HMF	Strong temporal associations between the onset of NEC and the initiation of enteral feeding or powdered cows milk-based fortification in preterm infants
	Brown, et al, 2020 (16)	Cochrane systematic reviews	18 trials	<37 weeks	Low- to moderate-certainty evidence showing that multi-nutrient fortifier increases in-hospital rate of weight gain, body length, or head circumference. Low-certainty evidence suggesting effects of multi-nutrient fortifiers on the risk of NEC in preterm infants	Fortification is associated with modest increases in in-hospital growth rates. Evidence is insufficient to show whether multi-nutrient fortification has any effect on long-term growth or neurodevelopment
Human milk-based	Ananthan, et al, 2020 (44)	Meta-analysis	1071	<37 weeks	Lower risk of NEC and weight gain in the human milk-based fortifier group. No differences for length, head circumference, and, in late-onset sepsis, mortality	Fortification with human milk-based fortifier compared with bovine milk-based fortifier decreased the risk of NEC but was associated with lower weight at 3 weeks of fortification or at discharge. The quality of evidence is low to very low, and adequately powered, well-designed RCTs free from industry bias are needed
	Cristofalo, et al, 2013 (45)	Multicenter RCT	53	27.5 vs. 27.7 weeks	Lower parenteral nutrition days (27 vs 36), lower incidence of NEC (3% vs 21%) and surgical NEC rate in human milk-derived fortifier versus bovine milk–based preterm formula	This trial supports the use of an exclusive human milk diet to nourish extremely preterm infants in the neonatal intensive care unit
	Hair, et al, 2016 (46)	Multi-center retrospective cohort study	1587	26.4 vs. 26.5 weeks	The human milk diet group had significantly lower incidence of proven NEC, mortality, late-onset sepsis, ROP, and BPD compared with the bovine-based diet group	An exclusive human milk diet offers manifold benefits and improves multiple clinical outcomes with the implementation of such a feeding protocol
	Asbury, et al, 2022 (48)	Triple-blind RCT	119	28.1 vs. 27.9 weeks	Infants fed a human milk-based fortifier had lower microbial diversity (Shannon index), and overall higher relative abundances of Proteobacteria and unclassified Enterobacteriaceae, as well as lower relative abundances of Firmicutes and Clostridium *sensu stricto*, compared with infants fed a bovine milk-based fortifier. A rapid decrease in the relative abundance of Enterococcus was also observed among human milk-based fortifier-fed infants during postnatal weeks 2–4, which plateaued thereafter	Different fortifiers alter the developing gastrointestinal microbiota of very low birth weight infants. Furthermore, dose-dependent relationships exist between individual enteral feed components and the gut microbial communities of very low birth weight infants
	O'Connor, et al, 2018 (49)	Blinded RCT	127	27.7 weeks	No statistically significant differences were identified in feeding interruptions, the mortality and morbidity index, changes in fecal calprotectin, or growth z scores between very preterm infants who received human milk-based fortifier and those who received bovine milk-based fortifier	Routine substitution of bovine milk-based fortifier with human milk-based fortifier for the improvement of feeding tolerance should not be set as a therapeutic target
	Jensen, et al, 2024 (50)	Multi-center RCT	228	22^+0^–27^+6^ weeks	Of the 115 infants assigned to human milk-based fortifier, 41 (35.7%) fulfilled the criteria of either NEC, sepsis, or death, compared with 39 (34.5%) of 113 infants assigned to bovine milk-based fortifier. Adverse events did not differ significantly between groups	The results do not support routine supplementation with human milk-based fortifier as a nutritional strategy to prevent NEC, sepsis, or death in extremely preterm infants exclusively fed human milk
	Reyes, et al, 2025 (51)	Systematic Review with Meta-Analysis	20 studies	25.5–29.8 weeks	In direct comparisons of fortifier type with a base diet of human milk, estimates suggested lower odds of Bell Stage ≥ 2 NEC by 35% (OR: 0.65; 95% CI: 0.44, 0.97; *p* = 0.03, n = 2102) and surgical NEC by 49% (OR: 0.51; 95% CI: 0.26, 0.98; *p* = 0.04; *n* = 1659) with human milk-derived fortifiers	The consistency of effect sizes across study designs suggests that the benefits of human milk-derived fortifiers are clinically meaningful
Preterm formula as fortifier	Gupta, et al, 2020 (52)	RCT	148	<34 weeks	The weight gain velocity was significantly better in the fortification group as compared to the standard group. The overall linear growth showed significant improvement in the fortification arm	Compared to unfortified human milk, fortification with infant formula results in better growth parameters and may represent a valuable alternative in low-resource settings
	Lin, et al, 2020 (53)	Retrospective and longitudinal study	101	27.3 vs 26.4 weeks	Significantly higher daily enteral milk intake in the first 4 weeks of life, and higher percentages of fortification in the human milk-fed infants in the first 8 weeks after birth. Also significantly increased in body weight growth in terms of z-score at term equivalent age and had better language and motor performance at 24 months old	Using concentrated liquid preterm formula as a strategic alternative fortification at the initial stage is feasible. It may be beneficial in improving postnatal growth and neurodevelopmental outcomes
	Chinnappan, et al, 2021 (54)	RCT	123	<34 weeks	The incidence of feed intolerance was lower in the preterm formula fortification group, and fewer neonates required withholding fortification for 24 h or more. No difference in weight gain and incidences of NEC stage II or higher and EUGR between the preterm formula fortification group and the human milk fortifier group	Fortification with preterm formula powder is not inferior to fortification with human milk fortifiers. Given the possible reduction in feed intolerance and lower costs, preterm formula might be a better option for fortification, especially in resource-restricted settings
	Aryadevi, et al, 2025 (55)	Meta-analysis	434	<34 weeks	No significant differences were found in the gain of weight, length, or head circumference between preterm formula and human milk fortifier. There were comparable risks of morbidities	Very low-certainty evidence suggests that fortification with preterm formula may be a safe alternative, with similar safety profiles and effects on growth
Donkey milk-based	Bertino, et al, 2019 (59) Cresi, et al, 2019 (60) Peila, et al, 2020-2024 (61–63)	RCT	156	<32 weeks	The risk of feeding intolerance and gastroesophageal reflux frequency tended to be lower in donkey milk-derived fortifier than in bovine-derived fortifier. No difference in risk was observed between the two groups for any of the considered allergy-related outcomes and long-term neurodevelopmental outcomes	Donkey milk-derived fortifier is a safe alternative to bovine fortifier, offering better feeding tolerance and less gastroesophageal reflux, without compromising growth or neurodevelopment
Bovine Colostrum as fortifier	Ahnfeldt, et al, 2023 (68) Kappel, et al, 2022 (70)	Multi-center RCT	232	26–31 weeks	BC infants received more protein than conventional fortifier infants and showed several elevated amino acid-levels. The incidence of bowel gas restlessness increased with laxative treatments and days of fortification in both groups, but laxatives were prescribed later in BC infants. With advancing age, stomach appearance scores improved, but more so in BC infants	It is feasible and safe to use BC as a fortifier but adjustment in protein composition and further micronutrient supplementation to the BC product may be necessary. BC induced similar or slightly improved bowel habits in preterm infants compared with conventional fortifier
	Ping, et al, 2026 (69)	RCT	146	26^+0^–31^+6^ weeks	No statistically significant difference was found in the incidence of feeding intolerance or in most of the nutritional or body growth parameters	BC appeared safe when used as a fortifier to human milk for very preterm infants with slow feeding advancement, but did not improve feeding tolerance or clinical variables

RCT, randomized controlled trial; BC, bovine colostrum; NEC, necrotizing enterocolitis; DERR, daily event rate ratio; EUGR, extrauterine growth restriction; ROP, retinopathy of prematurity; BPD, bronchopulmonary dysplasia.

Cow's milk-based fortifiers use mature cow's milk as their raw material. The insoluble casein in cow's milk accounts for approximately 80% of its protein content and exists in a micellar form (30). Under the influence of gastric acid, this micellar structure undergoes rearrangement or partial dissociation, forming a curd-like mass within the gastrointestinal tract, which delays gastric emptying (31–33). Cow's milk also contains *α*- and *β*-lactoglobulins, with the latter being absent in human milk and regarded as a primary allergen in cow's milk (34). Cohort and randomized controlled trial (RCT) studies have substantiated the efficacy of whole-protein supplementation (35–37). A Cochrane systematic review identified 18 RCTs in which a total of 1,456 preterm infants concluded that fortification with cow's milk-based fortifier increases growth rates of preterm infants during their first hospital admission but does not provide enough evidence to show any effects on longer-term growth or development (16). However, due to concerns regarding tolerability and allergenicity, manufacturers employ proteins with varying degrees of hydrolysis as protein sources. A pilot RCT suggested that preterm infants fed partially hydrolyzed protein formulas have shorter gastric emptying times, although intestinal absorption is reduced (38). In preterm infants with a family history of atopic dermatitis, premature exposure to cow's milk heightens the risk of allergy (39), whereas the use of hydrolyzed protein enhances preterm infant growth and reduces the risk of milk-protein sensitization (40). Nevertheless, a recent RCT study indicated that preterm infants supplemented with HMF or fed exclusively formula for four months post-discharge do not exhibit an increased risk of infantile allergic diseases compared to those exclusively breastfed (41). A Poisson regression analysis also associates the use of cow's milk-derived powdered human milk fortifiers with an elevated incidence of NEC (42). Although many professionals prefer hydrolyzed protein fortifiers, there is a lack of robust evidence supporting this practice.

To mitigate potential allergenic risks and reduce the incidence of NEC, exclusive human milk diets (EHMD, consisting of breast milk supplemented with human milk-derived fortifiers) present a viable alternative. Human milk fortifiers are derived from donated breast milk, which is pasteurized and standardized to preserve natural human milk proteins. By 2015, approximately one-fifth of NICUs utilized human milk-based fortifiers in the United States of America (9). Research suggests that exclusive human milk feeding may shorten the duration of parenteral nutrition (43), reduce the incidence of NEC (44), and may be associated with improved early growth, lower mortality rates, and a reduced incidence of late-onset sepsis, retinopathy of prematurity, and bronchopulmonary dysplasia (BPD), potentially having long-term positive health effects (45, 46). An observational study identified significant associations between fortifier type and the abundance of Bifidobacteria and Lactobacilli in the gut of preterm infants (47). Infants fed human milk-derived fortifiers exhibited lower microbial diversity and a more uniform microbial distribution, characterized by a greater abundance of the phylum Proteobacteria and family Enterobacteriaceae, along with a reduced abundance of the phylum Firmicutes and Clostridium spp (48). However, the pioneering OptiMoM RCT study (49)—the first to compare human milk fortifiers with cow's milk fortifiers in the complete absence of formula milk—found no differences between the two in terms of feeding tolerance, physical growth, or morbidity. The results of Jensen et al.'s RCT study (50) were largely consistent with findings from OptiMoM. A recent meta-analysis incorporating six RCTs and sixteen retrospective studies demonstrated that human milk-derived supplements significantly reduced both non-surgical and surgical NEC incidence: cases of Bell ≥2 severity decreased by 35% and surgical NEC cases by 49% (51). The effects of human milk-derived fortifiers on metabolism and body composition in preterm infants remain unclear and requires further investigation. Moreover, human milk-derived fortifiers are prohibitively expensive, necessitating a careful assessment of their benefit-risk ratio. Their limited availability also poses a significant constraint on their widespread global adoption (15).

In certain resource-constrained NICUs where conventional human milk fortifiers are inaccessible, preterm infant formula has been experimented with as a fortifier. In comparison to unfortified human milk, fortification with preterm formula facilitates linear growth in preterm infants without elevating complication rates (52). A quality improvement study revealed that employing concentrated liquid preterm formula, which has an energy density of 30 kcal/oz, as a substitute for powdered HMF might confer substantial advantages in promoting growth and neurodevelopmental outcomes (53). A subsequent non-inferiority RCT demonstrated that fortifying breast milk with preterm infant formula resulted in comparable weight gain, as well as similar incidences of NEC and EUGR to conventional fortifiers, while presenting lower feeding intolerance and reduced usage costs (54). The most recent meta-analysis indicated no disparities between groups in terms of preterm infants' weight, head circumference, and length gain, nor in complication rates; however, the level of evidence for these conclusions was extremely low, due to the included studies having some method concerns and a high risk of bias (55). It is noteworthy that formula fortification may lead to heightened osmolarity, inconsistent protein content, and contamination risks. The optimal fortification dosage remains unvalidated (56). Although its benefits and safety require further assessment, infant formula may serve as an alternative to conventional fortifiers when resources are scarce (54).

Another fascinating alternative is the donkey milk-derived fortifier. Donkey milk has a casein/whey protein ratio approaching that of human milk, is abundant in alpha-lactalbumin, lysozyme, and lactoferrin, and contains higher levels of immunoglobulin A, rendering it the closest substitute for human milk (57). Its protein and fat composition is more adaptable to neonatal requirements (58). In an RCT Donkey milk-derived fortifier, enhanced feeding tolerance while achieving comparable physical growth outcomes compared to cow milk-derived fortifiers with equivalent calories and protein (59). Subsequent additional data analysis revealed that the use of donkey-derived human milk fortifier reduced the gastroesophageal reflux frequency in preterm infants with feeding intolerance (60). Analysis of follow-up data from the same study revealed donkey milk-derived fortifier does not impact the development of allergic manifestations in the first years of life, as well as similar neurodevelopmental and auxological outcomes at 18 months of age (61–63). Nevertheless, the limited availability of donkey milk constrains its extensive use, and the current evidence is inadequate to support its routine application.

Bovine colostrum (BC) refers to the milk produced by cows in the initial few days post-calving. Beyond its nutritional value, it is replete with immunoglobulins, bioactive peptides, and growth factors, exhibiting multiple biological functions, including stimulating cell growth, combating infections, and regulating immunity (64). Extensive research on preterm animals has shown that BC, whether used as exclusive feeding or supplementation, plays a pivotal role in nutritional and immune support, growth and development, and gut health (64). In preterm pigs fed donor human milk, BC fortifiers demonstrated superiority over conventional fortifiers in supporting growth, intestinal function, nutrient absorption, and mucosal defense (65, 66). But recently, two multi-center RCT studies of BC in Denmark and China found no improvement in feeding tolerance or other clinical variables when BC was used as a fortifier, with minimal impact on gut microbiota (67–69). BC induced similar or slightly improved bowel habits in preterm infants (70). These limited clinical studies on preterm infants suggest that BC fortification is safe. However, further research is required to confirm the significant effects observed in animal studies, and understand better the optimal timing, dosage, and duration of BC administration.

### 3.2 Classification based on milk form

Human milk fortifiers are classified according to their final product form into liquid and powdered types (Table 2). Liquid fortifiers commonly exist as concentrated solutions and are subjected to sterilization procedures (e.g., pasteurization) to guarantee safety. Powdered fortifiers are mainly prepared through spray-drying or freeze-drying methods. An *in vitro* experiment suggests that powdered fortifiers are not completely sterile, and their use may pose a risk of infection with Cronobacter sakazakii (71). Most recently, seven preterm infants were reported as a cluster of acute gastrointestinal illness, temporally associated with a single batch of powdered HMF contaminated with Bacillus aceticus in India (72). Bacillus cereus spores survive pasteurization and drying and can germinate after ingestion, releasing enterotoxins that cause diarrheal illness, especially in preterm infants (73, 74). Liquid fortifiers utilize aseptic filling techniques, which facilitate accurate dosing and mitigate contamination risks (75). Considering their better ability to maintain sterility compared to powdered fortifiers, the US Food and Drug Administration strongly advocates the use of liquid products over powdered ones in NICU to minimize contamination and infection risks (76).

**Table 2:** Clinical studies on human milk fortifiers of different in liquid form.

Reference	Research method	Sample size	Gestation age	Result	Conclusion
Lainwala, et al, 2017 (78)	Retrospective study	67	29.2 vs. 29.6 weeks	Acidified liquid HMF group had significantly higher incidence of metabolic acidosis and lower bicarbonate and base excess levels relative to infants receiving heat treated liquid HMF	Acidified liquid HMF was associated with an increased incidence of metabolic acidosis
Schanler, et al, 2018 (79)	RCT	164	≤32 weeks	Infants fed non-acidified liquid human milk fortifier had significantly greater weight gain from study days 1 to 15. Infants fed acidified liquid human milk fortifier received more protein yet had lower blood urea nitrogen values, also had more vomiting, gastric residuals, metabolic acidosis, and abdominal distension	Infants fed an acidified liquid HMF had higher rates of metabolic acidosis, poorer feeding tolerance, and poorer initial weight gain
Cordova, et al, 2021 (80)	Retrospective cohort study	255	<33 weeks	Infants receiving acidified HMF had higher adjusted odds of metabolic acidosis than infants receiving non-acidified HMF. There were no differences between groups in size z-scores at discharge	Fortification with a liquid acidified HMF may induce metabolic acidosis in the first month of life compared with fortification with a liquid non-acidified HMF; may not affect growth
Moya, et al, 2012 (82)	Multi-center RCT	150	≤30^+3^ weeks	The liquid HMF had a significantly higher achieved weight, length, head circumference, and linear growth rate than the powder HMF. No differences in measures of feeding tolerance or days to achieve full feeding volumes. Prealbumin, albumin, and blood urea nitrogen were higher in the liquid HMF group than the control group. No difference in the incidence of confirmed sepsis or NEC	The use of a new liquid HMF in preterm infants is safe and has benefits of improved growth and the avoidance of powder product use

RCT, randomized controlled trial; HMF, human milk fortifier; NEC, necrotizing enterocolitis.

Powdered HMF formulations generally contain higher concentrations of most nutrients than liquid HMF. Nevertheless, the disparity between fortified human milk and the recommended intake levels is comparable regardless of whether liquid or powdered formulations are employed (29). A common disadvantage with liquid HMF is displacement, as it takes up more than 15% of the available space, thereby reducing the amount of human milk (29). Some liquid fortifiers undergo acidification followed by heat treatment during the preparation process (77), which reduces the pH of the fortifier, leading to decreased bicarbonate and alkaline reserve levels (78) and a notably elevated incidence of metabolic acidosis (79, 80). RCT study have also observed that acidification may also induce protein precipitation and diminished lipase activity, thereby influencing nutrient bioavailability (81).

### 3.3 Classification by component

Human milk fortifiers can be classified into multi-component and single-component formulations according to their constituent elements. Multi-component fortifiers are the most commonly employed in clinical practice, supplying supplementary nutrients to human milk, including proteins, calcium, phosphates, carbohydrates, vitamins, and trace minerals (83). Single-component fortifiers that contain only proteins, lipids, or carbohydrates are also accessible in the market (15). These are generally employed as supplements to multi-component fortifiers during individualized fortification or can be added separately to human milk (36, 37, 84).

#### 3.3.1 Multi-component fortifiers

##### 3.3.1.1 Protein

Multi-component fortifiers have been employed in clinical practice for several decades, and their compositions have been continuously optimized in accordance with clinical requirements (Table 3). Nevertheless, they still fall short of meeting all the recommended intakes of macronutrients and micronutrients (14). When energy intake is sufficient, protein intake serves as the primary determinant of lean body mass growth (85). Historically, a high protein intake in preterm infants was regarded as perilous. Research indicated that infants fed with a higher-protein (6.0–7.2 g/kg/day) formula had significantly lower IQ scores compared to those fed with a low-protein formula (86). To avoid excessive protein intake, most multi-component fortifiers maintained protein levels at low concentrations ranging from 1.0 to 1.1 g/dL. Assuming that human milk has a consistent protein content and energy density, standard fortification led to a considerable proportion of preterm infants having inadequate protein intake (87). In fact, the protein content of breast milk decreases significantly as the lactation period progresses (10, 19). Consequently, the utilization of high-protein fortifiers (≥1.4 g/100 mL and above) has been gradually increasing (88). Several RCTs and cohort studies have confirmed that high-protein fortifiers facilitates infant physical development (36, 82, 89–91), shows good tolerability (36), and elevates neutrophil and lymphocyte counts as well as serum IgA concentrations, thereby enhancing immune function (92). A recent meta-analysis revealed that preterm infants fed with high-protein fortifiers gained slightly more weight during hospitalization compared to those receiving medium-protein fortifiers (1–1.4 g/100 mL), while those fed with low-protein fortifiers (<1 g/100 mL expressed breast milk) exhibited lower gains in weight and length. However, the current evidence is insufficient to evaluate the influence of protein content on adverse reactions or long-term outcomes such as neurodevelopment. Some commercially available liquid fortifiers can increase the protein content to 1.8 g/100 mL, thereby raising the protein intake of preterm infants to satisfactory levels (82).

**Table 3:** Commonly used multi-component human milk fortifiers (HMF)^a^.

Nutrient	Cow Milk-based HMF						Human Milk-based HMF				BC
HMF	A ^L^	B ^P^	C ^P^	D ^P^	E ^P^	F ^P^	G ^L^	H ^L^	I ^L^	J ^L^	K ^P^
Energy, kcal	7	4.88	4.35	3.89	3.47	3.48	29.4	43.7	57.7	71.5	4.73
Protein, g	0.5^EH^	0.387^PH^	0.355^PH^	0.28^W^	0.25^EH^	0.2^EH^	1.2	1.8	2.4	3.0	0.5
type	casein/whey	casein/whey	whey	whey	casein	whey	-	-	-	-	-
Fat, g	0.21	0.35	0.181	0.1	-	0.004	1.9	2.9	3.8	4.7	0.212
Carbohydrate, g	0.75	0.07	0.324	0.5	0.62	0.67	1.9	2.7	3.4	4.1	0.204
Iron, mg	0.11	0.51	0.45	0.1	-	0.34	-	-	-	0.1	<0.01
Zinc, mg	0.31	0.25	0.24	0.28	0.14	0.18	1.2	1.2	1.3	1.6	0.076
Calcium, mg	30	31.7	18.9	32.5	15	15	104	108	110	142	9.2
Vitamin A, IU	197	335	277	172	175.5	236.4	38.3	46.1	70.9	76.9	33.03
Vitamin D, IU	35	52.8	35.2	33.3	46	30	0	4	4	4	<0.1
Vitamin K, μg	2.4	1.54	1.88	2.3	1.44	1.6	-	-	-	-	-

a Nutrient composition per 1 g of the powdered fortifier. P, powder; L, liquid; PH, partially hydrolyzed; EH, extensively hydrolyzed. HMF: A, Abbott Similac (liquid) (5 mL); B, Mead Johnson Enfamil; C, Nestlé PreNAN FM85; D, Abbott Similac; E, Aptamil (FMS); F, Nestlé NAN FM85; G, Prolact+4 (20 mL); H, Prolact+6 (30 mL); I, Prolact+8 (40 mL); J, Prolact+10 (50 mL). Nutritional information is presented on the product label or the official website.

##### 3.3.1.2 Energy

The energy requirements of preterm infants increase as their weight gains due to the accumulation of fat stores (93). Multi-component HMFs, while fortifying protein levels, also augment the energy density of human milk by approximately 20% through the supplementation of carbohydrates and fats (94). However, the macronutrient ratios vary among different products, with some fortifiers having a higher proportion of carbohydrates and others a higher fat content. Currently, the human milk fortifiers on the market are characterized by elevated lipid content and reduced carbohydrates, thus lowering the osmolarity of fortified human milk (95, 96). Moreover, lipids serve as a source of essential fatty acids (EFAs), which can improve the EFA status of preterm infants (97).

##### 3.3.1.3 Calcium, phosphorus, iron

Preterm infants exhibit low skeletal reserves of calcium and phosphate at birth. Meanwhile, breast milk contains relatively low concentrations of calcium and phosphate, measuring 25–27 mg/100 mL and 14–16 mg/100 mL, respectively (98). Nevertheless, the recommended intakes of calcium and phosphate for very low birth weight infants are 150–220 mg/kg and 75–149 mg/kg, respectively (99). The use of multi-component fortifiers has alleviated mineral deficiencies (100). Novel human milk fortifiers incorporating highly soluble calcium glycerophosphate may augment calcium retention (101). There are substantial disparities in iron content among different multi-component fortifiers. *In vitro* experiments, Iron-containing fortifiers attenuate the bacteriostatic effect of breast milk against Escherichia coli (102) and reduce its antimicrobial activity (103–105). However, other experiments have revealed no influence of iron content on antimicrobial activity (106, 107). Prolonged clinical experience has not indicated adverse effects resulting from iron fortification. Consequently, the dosage of oral iron supplements should be adjusted according to the iron content of the fortifiers consumed.

##### 3.3.1.4 Vitamins

Vitamin A, a critical micronutrient, exerts a significant influence on infant health. The retinyl esters present in human milk fortifiers necessitate hydrolysis within the gut prior to absorption. It is advised that very low birth weight infants undergo oral supplementation with 1,200 μg/kg/day of retinyl esters commencing from the initiation of full enteral feeding until discharge. The use of human milk fortifiers containing 600 μg/kg/day has been shown to enhance plasma retinol levels at the time of discharge; however, these levels may still be suboptimal Consequently, future trials should investigate augmenting the retinyl ester content in HMFs (108). All neonates require supplementary vitamin K1 to prevent vitamin K deficiency. Human milk-derived fortifiers exhibit significantly lower vitamin K1 levels compared to cow’s milk-based preparations (109). In the absence of supplementary intake, infants receiving an exclusive human milk diet demonstrate notably lower dietary vitamin K1 intakes than the recommended levels. Therefore, an adequate and sustained dietary vitamin K intake is of critical importance for preterm infants consuming human milk-derived fortifiers (110, 111).

Vitamin D is an indispensable micronutrient for bone health. Current oral intake recommendations stand at 200–400 IU/day as per the American Academy of Pediatrics (AAP) (112), whereas the European Society for Paediatric Gastroenterology, Hepatology, and Nutrition recommends 800–1,000 IU/day (21). Not all fortifiers provide the amount recommended by the AAP, and certainly not in accordance with the ESPGHAN recommendation (113). Nevertheless, when vitamin D-containing preterm infant formula, human milk fortifiers, and additional vitamin D supplements are used simultaneously, the actual intake may surpass the recommended levels. Hence, meticulous calculation of cumulative doses is essential when utilizing vitamin D-containing fortifiers (114).

#### 3.3.2 Single-component fortifiers

Protein fortifiers, predominantly sourced from cow's milk, have been commercially accessible in some countries for several years (Table 4). However, they were not specifically formulated for preterm infants (36, 37, 86, 115). A novel protein fortifier specifically developed for preterm infants integrates partially hydrolyzed protein sources (116). Protein fortifiers play a vital role in attaining individualized nutritional fortification. Even subsequent to the standard fortification of preterm human milk utilizing multi-component fortifiers, a substantial proportion of infants fail to reach the protein intake levels and protein-to-energy ratios recommended by guidelines. Consequently, beyond standard fortification, adaptive fortification makes adjustments by adding varying dosages (0.4–0.8–1.2 g) of protein fortifier in accordance with the individual preterm infant's amino acid metabolism (blood urea nitrogen levels) (35). Targeted fortification entails periodically analyzing the composition of human milk to ascertain its actual protein content relative to the ideal breast milk protein target (2.7–3.3 g/100 mL) and then supplementing with additional protein beyond standard fortification to achieve the target (35). Both methods have demonstrated by multiple RCT studies significant improvements in protein intake and promotion of physical growth in the short term (35–37, 84, 117).

**Table 4:** Common used protein fortifiers^a^.

Nutrient	Beneprotein	Instant protein	Aptamil (PS)	Preemie
Energy, *kcal*	3.6	3.8	3.4	3.6
Protein, *g* *type*	0.86 ^W^, whey	0.91 ^W^ whey	0.82 ^EH^ casein/whey	0.72 ^EH^^b^
Fat, *g*	0.01	0.01	-	-
Carbohydrate, *g*	0.04	0.006	-	-
Sodium, *mg*	2.1	0.15	7.8	8.2
Potassium, *mg*	-	0.15	-	-
Calcium, *mg*	-	14.5	5.2	12.8
Phosphorus, *mg*	-	8	5.2	0.73

a Nutrient composition per 1 g of the powdered human milk fortifier (HMF).

b No information provided. Nutritional information is presented on the product label. PH, partially hydrolyzed; EH, extensively hydrolyzed;W,whole protein. .

Carbohydrate supplements predominantly comprise maltodextrin, a glucose polymer (94), which includes monosaccharides, disaccharides, oligosaccharides, or polysaccharides. These carbohydrates can be sourced from cow's milk, human milk, or soy milk or be synthetically manufactured (118). Fat supplements mainly consist of medium-chain triglyceride emulsions, long-chain fatty acid emulsions (15), and a newly developed human milk-derived cream supplement (119) designed to enhance the energy density of human milk. When the energy content of preterm infant human milk or donor milk is below 67 kcal/mL, a 2.5 kcal/mL fat supplement can be added (120). RCTs study by Hair et al. found that preterm infants administered this supplement exhibited accelerated weight and length gain, along with marginally earlier discharge times, a tendency that was especially evident in those with concurrent BPD (119, 120). It is crucial to emphasize that when using single-component fortifiers for supplementation, caution must be exercised to avoid disturbing the appropriate protein-to-energy ratio.

## 4 Conclusions and recommendations for future research

The optimal scenario for HMFs is to provide each preterm infant with appropriate energy and nutrient provisions. Based on clinical circumstances and resource availability, HMFs of diverse origins, forms, and compositions are clinically employed, each possessing its own merits and limitations. Currently, powdered fortifiers of bovine origin are the most widely used. Regardless of cost considerations, human milk-derived and liquid fortifiers may have slight advantages on the risk of NEC. A major disadvantage of adding liquid fortifiers is that they replace more than 15% of the volume of mothers own milk. Single-component fortifiers are often used as supplements to multi-component fortifiers. There is currently no clear evidence indicating which type of fortifier is optimal. It may be unrealistic to anticipate a single ideal HMF that fully satisfies the nutritional requirements of preterm infants without augmenting adverse effects, particularly since factors beyond the type of fortifier, such as timing and administration methods, also exert an influence.

According to the data, individualized fortification, including adjustable fortification and targeted fortification, might be a more effective fortification than standard fortification (121). These approaches dynamically adjust the use and dosage of various fortifiers based on real-time metabolic indicators and point-of-care human milk analysis (122), thereby achieving more precise nutritional delivery, a reduced risk of under- or over-nutrition, improved growth rates, and potentially better neurodevelopmental outcomes (36, 116). However, several barriers hinder its widespread use: the need for frequent blood sampling or costly near-infrared milk analyzers, increased clinical workload, a lack of standardized protocols, and concerns regarding the reliability of rapid testing methods (15).

Future research should focus on developing more convenient and effective personalised fortification methods to ensure precise nutritional intake in preterm infants, thereby promoting their physical growth and improving long-term outcomes. Concentrating human milk may be a promising future fortification strategy as it reduces water content through osmosis without the addition of exogenous fortifiers (123), thereby increasing nutritional density and avoiding potential fortifier-related side effects. However, uncertainties remain regarding this method, including limited technical feasibility in routine clinical settings, risk of bacterial contamination, loss of heat-labile components during heat-based concentration, lack of commercial equipment and insufficient evidence regarding its safety and effects on growth outcomes (124). Further research is needed to determine whether human milk concentration can act as a safe and effective alternative to, or supplement for, conventional human milk fortifiers.
